# The Electrocardiogram‐Based Phase Adjustment Task (PAT‐ECG): An Open‐Access Laboratory Test of Cardiac Interoceptive Accuracy

**DOI:** 10.1111/psyp.70415

**Published:** 2026-09-30

**Authors:** James Hodgetts, Germano Gallicchio, Shaun Mckiernan, Christopher Ring, Jennifer Murphy, Andrew Cooke

**Affiliations:** ^1^ School of Sport Science, Institute for the Psychology of Elite Performance (IPEP) Bangor University Bangor UK; ^2^ School of Sport, Exercise and Rehabilitation Sciences University of Birmingham Birmingham UK; ^3^ School of Psychology University of Surrey London UK

**Keywords:** cardiac interoception, electrocardiogram, heartbeat perception, interoception, interoceptive accuracy, phase‐adjustment task

## Abstract

Interoceptive accuracy plays a central role in theories of emotion, and atypical interoceptive accuracy is increasingly recognized as a feature in the pathophysiology of multiple clinical and subclinical conditions. However, existing methods for assessing interoceptive accuracy have limitations that may constrain research progress. This study presents the first electrocardiogram‐based implementation of the phase adjustment task (PAT‐ECG), a rigorous measure of cardiac interoceptive accuracy, and provides the materials and experimental code for its use. Similar to existing PAT implementations, the task presents tones at each participant's heart rate and requires them to adjust the phase of the tone to synchronize it with their heartbeats. Crucially, this implementation replaces the photoplethysmographic signal used in previous implementations with ECG, enabling superior temporal resolution and more precise triggering via the R‐wave peak. We additionally introduce a novel phase‐delay timing procedure during task execution and explore new data‐analytic approaches. Data from 91 participants are used to demonstrate the task, and eight options for classifying individuals as interoceptive or non‐interoceptive are evaluated. Seventeen percent of participants were classified as interoceptive at rest via PAT‐ECG. Findings extend evidence for distinct variable‐period and fixed‐period interoceptive pathways. Weighting trials by confidence ratings further influenced interoceptive accuracy scores. The original PAT represented a significant advancement in cardiac interoceptive accuracy assessment; this open‐access ECG‐based implementation extends PAT methodological precision, providing an even more rigorous assessment tool for further advancing interoception theories and research.

## Impact Statement

Accurate measurement remains a central challenge in interoception research. This study advances the field by introducing the first electrocardiogram‐based phase adjustment task (PAT‐ECG), offering superior precision to existing PAT implementations alongside novel implementation and analytic options. Findings extend evidence for distinct interoceptive pathways and highlight how analytic choices shape classification outcomes. PAT‐ECG is provided as an open‐source tool to support the next generation of rigorous and precise interoception research.

Interoception, which refers to the perception of the body's internal state (Craig 2002), has long been viewed as a core element in theories of decision making and emotion (Damasio 1999; James 1884). Beyond its central role in emotion regulation (Kever et al. 2015), atypical interoception has been proposed as an underlying feature in the pathophysiology of many clinical and subclinical conditions including anxiety, alexithymia, panic disorder, eating disorders, alcohol and substance abuse (Khalsa et al. 2018; Murphy, Catmur, and Bird 2018; Naqvi and Bechara 2010; Paulus and Stein 2010; Paulus and Stewart 2014; Schaefer et al. 2012). It is therefore unsurprising that interoception is an increasingly popular concept to study (Ritz et al. 2024). However, while the theorized links between interoception and various psychological conditions and processes related to emotion and decision making are compelling, empirical support for these associations is mixed. Inconsistent findings have prompted ongoing debate regarding both the magnitude of associations between interoception and psychological constructs and the validity of some commonly used interoception assessments (e.g., Desmedt et al. 2023; Trevisan et al. 2019; Murphy 2024; Zamariola et al. 2019). To precisely study any concept, it is imperative to have rigorous methods of assessment. This study presents the electrocardiogram‐based implementation of the phase adjustment task (PAT‐ECG), a rigorous test of cardiac interoceptive accuracy, and outlines the materials and experimental codes for its use.

## Cardiac Interoceptive Accuracy: Current Tests

The tight coupling between the heart and the autonomic nervous system, and their central roles in emotion (e.g., Kreibig 2010) and responses to stress (e.g., Porges 1995), make cardiac signals particularly relevant for interoception researchers. Individual differences in cardiac interoceptive accuracy have been linked to emotional reactivity and regulation, as well as to psychological conditions characterized by emotion dysregulation (e.g., Braet and Braet 2024; Murphy, Brewer, et al. 2018), although the consistency and magnitude of these associations remain debated and may depend, in part, on the interoception assessment employed (Ferentzi et al. 2019; Ring and Brener 2018). Nevertheless, cardiac signals have become the predominant focus of interoceptive accuracy research, with studies typically employing tasks designed to index individuals' ability to perceive their own heartbeat sensations (Murphy 2024).

The two most common tasks to test cardiac interoceptive accuracy are heartbeat counting and heartbeat detection. Heartbeat counting paradigms ask participants to count or estimate heartbeats in a few short (typically < 1 min) intervals (e.g., Schandry 1981). Interoceptive accuracy is inferred from the correspondence between the actual and the reported number of heartbeats. Meanwhile, two‐interval heartbeat detection paradigms ask participants to judge over many trials the simultaneity between their heartbeat sensation and an exteroceptive stimulus (e.g., tone) provided either at a time point when the heartbeat is assumed to be perceived (e.g., 128 ms after the onset of left ventricular contraction, marked by the R‐wave of the electrocardiogram—which is classified a priori as a synchronous trial) or at time not typically associated with heartbeat perception (e.g., 384 ms after the R‐wave—an asynchronous trial) (e.g., Whitehead et al. 1977). Interoceptive accuracy is inferred from a score based on the relative number of correct judgments (see Brener et al. 1993).

Despite their popularity, the validity of the scores yielded from both tasks has been questioned (for review see Brener and Ring 2016; Desmedt et al. 2023). In brief, performance on heartbeat counting tasks can be impacted by beliefs about heart rate irrespective of whether a heartbeat was perceived. Accurate knowledge of one's heart rate can inflate task performance, potentially leading to mislabelling of poor or non‐heartbeat perceivers as highly interoceptive (Ring and Brener 1996; Ring et al. 2015). Meanwhile, a primary limitation of heartbeat detection paradigms is their difficulty in accounting for individual differences in the delay between the heartbeat stimulus and the perception of heartbeat‐tone synchrony. Extensions of the original two‐interval heartbeat detection paradigm have presented exteroceptive stimuli at multiple fixed delays relative to the electrocardiogram's R‐wave (e.g., R‐wave +0, +100, +200, +300, +400, +500 ms) and infer accuracy from the consistency of participants' selections. However, individual differences in factors such as the body location where the heartbeat is perceived, pulse arrival time, and ability to judge stimulus simultaneity can influence people's sensitivity to heartbeat‐tone synchronicity (for a discussion see Brener and Ring 1995; Palmer et al. 2025). Moreover, at least 20 trials per delay are recommended to yield reliable estimates in six‐interval tasks (Brener and Ring 2016; Kleckner et al. 2015), with more trials recommended for two‐interval tasks. It is worth noting that these suggested minimum trial number recommendations are far less than typically employed in standard psychophysical methods. The requirement for so many trials may increase the chances of individual differences in attention, fatigue or motivation influencing results, especially for methods that use over 100 trials. For a fuller discussion of these tasks and their respective limitations see Brener and Ring (2016) and Desmedt et al. (2023).

## A Need for New Approaches—The Phase Adjustment Task

To address some of these methodological issues, Plans et al. (2021) developed the Phase Adjustment Task (PAT). In brief, Plans et al.'s (2021) PAT presents participants with tones at their heart rate but with a variable delay after their heartbeat. It estimates the timings of heartbeats from a finger pulse signal captured via photoplethysmography (PPG) using a mobile‐phone camera. This is interfaced with a smartphone application that instructs users to turn an on‐screen dial to adjust the heartbeat‐to‐tone delays to match the timing of their heartbeat sensation. Users are encouraged to sample multiple delays until they feel that the tones are synchronous with their heartbeat sensations. They then press on the touchscreen to register their preferred delay for that trial. After completing multiple trials, the chosen delays are transformed into angles to represent their timings relative to heart period (i.e., the duration of the cardiac cycle). The consistency of the chosen angles across trials is then computed on a continuum from 0 (no consistency, selected delays are uniformly distributed) to 1 (perfect consistency, stable bias to a particular angle is evident in the delay selections) as the mean resultant length of the angles.

On the assumption that individuals perceive their heartbeats consistently in the same phase of the cardiac cycle (detected by analyses where each delay is expressed relative to the heart period of the same trial) or consistently at a similar delay from onset of the cardiac contraction (detected by analyses where all delays are expressed relative to the same heart period across trials) (see Palmer et al. 2026), above chance performance (e.g., interoceptive accuracy) can be inferred from the 0–1 consistency scores. Specifically, individuals who consistently prefer tones at similar angles across trials will achieve scores closer to 1 (i.e., superior interoceptive accuracy performance), whereas those who are unable to perceive their heartbeats are expected to choose inconsistently (randomly) across trials and thereby yield scores close to 0 (Palmer et al. 2026; Plans et al. 2021).

Importantly, because PAT scores are based on the consistency of judgments, so long as participants focus on a consistent body location (e.g., chest) when monitoring their heart beats over successive trials, PAT scores can tolerate some individual differences in perception (e.g., individual differences in the location of the heartbeat sensation and/or pulse arrival time). Further, simulation‐modeling work reported in detail by Plans et al. (2021) and Todd et al. (2024), and subsequently expanded by Palmer et al. (2026), demonstrated that 15–20 PAT trials were sufficient to distinguish reliably delay choices expected from a somewhat consistent responder from those that could plausibly occur by chance, with the benefits of additional trials only becoming apparent at 100+ trials. Given that the modeled reliability benefits associated with 100+ trials would likely be negated in practice by fatigue‐related threats to validity, the ability to obtain reliable information from 15 to 20 trials ensures that the PAT can deliver robust data without imposing excessive demands on participants.

## The Phase Adjustment Task—Strengths and Challenges

In addressing the key limitations of heartbeat counting and heartbeat detection tasks, the PAT sets an important new standard in cardiac interoceptive accuracy assessment. Further, by using a mobile‐phone camera to record the pulse, the original implementation of the task (i.e., Plans et al. 2021; Palmer et al. 2026) also benefits from allowing mobile and accessible recordings to facilitate large samples, repeat assessments, and the study of hard‐to‐reach groups.

However, while these benefits are clear, the precision of camera‐based PPG measures of heartbeat, compared to gold‐standard laboratory measures of the heartbeat via electrocardiogram (ECG), remains a source of debate. Some studies report strong agreement between pulse and ECG signals (Morelli et al. 2018). Others highlight limitations of PPG when compared to ECG, including lower signal‐to‐noise ratios, difficulty detecting discrete cardiac events due to the PPG wave's curved shape, and individual differences in waveform characteristics influenced by skin color, thickness, and adiposity (Fine et al. 2021; Quigley et al. 2024). It is acknowledged that smartphone‐based PPG using broadband (white‐light) illumination enables access to multi‐wavelength (RGB) information that may improve robustness to artifacts compared with single‐wavelength PPG typically used by wearable PPG sensors, especially when the heartbeat (as opposed to precise individual features of the cardiac cycle) is the sole feature of interest (Liu et al. 2014; Meier et al. 2024). Notwithstanding, it is intuitive that the ECG can provide a more precise assessment of cardiac activity, with a sharp peaked and reliable R‐wave to determine the timing of left ventricular contraction, clearly marking the end of one cardiac cycle and the beginning of the next.

Relatedly, finger‐based pulse measures, as used by the original implementation of the PAT, and many other cardiac interoception tests (e.g., Betka et al. 2018; Ewing et al. 2017; Herman et al. 2019), may risk artificially augmenting interoception scores where the handheld device applies pressure to the finger or the hand (Desmedt et al. 2023). Whilst evidence is not entirely consistent with this proposal—previous evidence has found phone‐based PPG to result in lower interoceptive accuracy scores when compared to both the hard‐clip pulse oximeter and ECG (Murphy et al. 2019)—individual differences in the amount of pressure applied to the phone sensor and in the sensitivity to pulsatile sensations at the fingertip could conceivably influence PAT performance. It is also conceivable that maintaining positioning of the finger over the camera and flash during the task may be distracting. Accordingly, while few experimental comparisons of the different methodologies exist, it is possible that cardiac interoceptive accuracy during any tests that use PPG may be artificially augmented by the pulse recording methodology. In light of these possible issues regarding Plans et al.'s (2021) implementation of the PAT, the development of an ECG‐variant of the task would offer a valuable solution for researchers seeking a precise laboratory‐based cardiac interoceptive accuracy assessment.

The original PAT recently received some updates by the original team to replace its prediction algorithm that estimated the start *and* end of each cardiac cycle based on 3 s epoch recordings of the finger pulse (Palmer et al. 2026). As outlined by their recent paper (Palmer et al. 2026), the accuracy of the original approach could be influenced by individual differences in heart rate variability. Their update to Plans et al.'s (2021) original PAT method proposes a solution to this issue via implementing continuous beat‐by‐beat finger pulse detection (Palmer et al. 2026). The revised method is ripe for use with continuous R‐wave to R‐wave detection via ECG.

Finally, Palmer et al. (2025) pooled data from six studies employing either the original (four studies) or updated (two studies) PAT and identified distinct patterns of interoceptive responding. One group of individuals showed temporal consistency in their selected perceptions of pulse‐tone synchrony. This pattern is consistent with accounts in which heartbeat perception is anchored to the detection of a mechanical event associated with the arrival of the blood pressure pulse, whose timing at a given bodily location is relatively stable across cardiac cycles (Ring and Brener 1992). In contrast, a separate subset of participants exhibited consistency in the phase angle of their selected delays, such that the absolute timing of the perceived pulse shifted proportionally with beat‐to‐beat fluctuations in cardiac cycle length (e.g., a 100 ms delay at a 1000 ms cardiac period increasing to 120 ms at a 1200 ms period). This dissociation between delay‐based and phase‐based consistency suggests heterogeneity in the temporal reference frames underlying heartbeat perception.

Importantly, although both delay and phase‐based responders could be identified offline, the original PAT implementations schedule tones at fixed absolute delays (in milliseconds) following each pulse and convert these delays into phase angles offline. As such, whereas capable of detecting both forms of interoceptive responding, the original fixed delay approach to scheduling may reduce sensitivity to phase consistent responding, particularly where variability in cardiac cycle length is high. The development of a complementary PAT‐ECG implementation that allows tones to be scheduled according to phase angle within the cardiac cycle (i.e., phase locked timing) can resolve this issue and optimize the detection of phase‐based responders. This development is theoretically and methodologically warranted.

## A New Implementation: The Electrocardiogram‐Based Phase Adjustment Task (PAT‐ECG)

To address potential limitations of the original implementation of the PAT (Plans et al. 2021), the present study reports on a new implementation of this task, the PAT‐ECG. It uses the R‐wave from high‐resolution ECG hardware (2500 Hz sampling rate) to determine the cardiac cycles used to generate tones and assess task performance. This enables detection of temporal differences between the heartbeat and auditory tones with sub‐millisecond precision, exceeding the temporal precision achievable with smartphone‐based PPG implementations (typically 30–60 Hz). Importantly, we also developed existing PAT implementations by introducing the option to present tones in phase angles within the cardiac cycle (i.e., their relative delay from an R‐wave) *during* data collection. Finally, we present an accompanying exteroceptive control task designed to be completed before the PAT‐ECG, and we describe analytic approaches to aid data interpretation. In the next section, we describe the development of the PAT‐ECG and share resources openly to facilitate its use. We then report task demonstration data of our novel phase angle variant of the PAT‐ECG from a large participant sample.

## 1 Method

### 1.1 Overview

Since this manuscript primarily offers a methodological contribution, the Method section is organized into two main subsections. First, we describe the development of the new implementation of the PAT, including design, trial structure, measurement principles, and open‐source resources to facilitate its use. Second, we present an empirical demonstration of the task in a sample of participants (*N* = 91) to illustrate some approaches to interoceptive classification using the PAT‐ECG.

### 1.2 Task Development

#### 1.2.1 Apparatus & Task

##### 1.2.1.1 PAT‐ECG

A first key feature of our implementation of the PAT is the replacement of the PPG signal used in the original implementation with an ECG signal to record cardiac activity. This crucial development allows precise measurement of the heartbeat to generate the tones used in the task and the precise detection of the chosen delays relative to a measured R‐wave. Participants must first be instrumented for ECG monitoring and real‐time detection of the R‐waves. The timing of the R‐waves is sent as input to bespoke PAT‐ECG hardware. This is briefly comprised of a 13‐bit optical Gray code encoder interfaced with a Teensy 4.0 microprocessor board and a Windows PC via USB and 25‐way D‐type connectors. The optical encoder and dial used by participants is illustrated in Figure 1A. A maker's kit for the PAT‐ECG hardware and the PAT‐ECG acquisition software, programmed in Arduino, are available at the PAT‐ECG GitHub repository (https://github.com/Shaunmac68/PAT‐ECG) and via Zenodo (https://doi.org/10.5281/zenodo.17063594).

**FIGURE 1:**
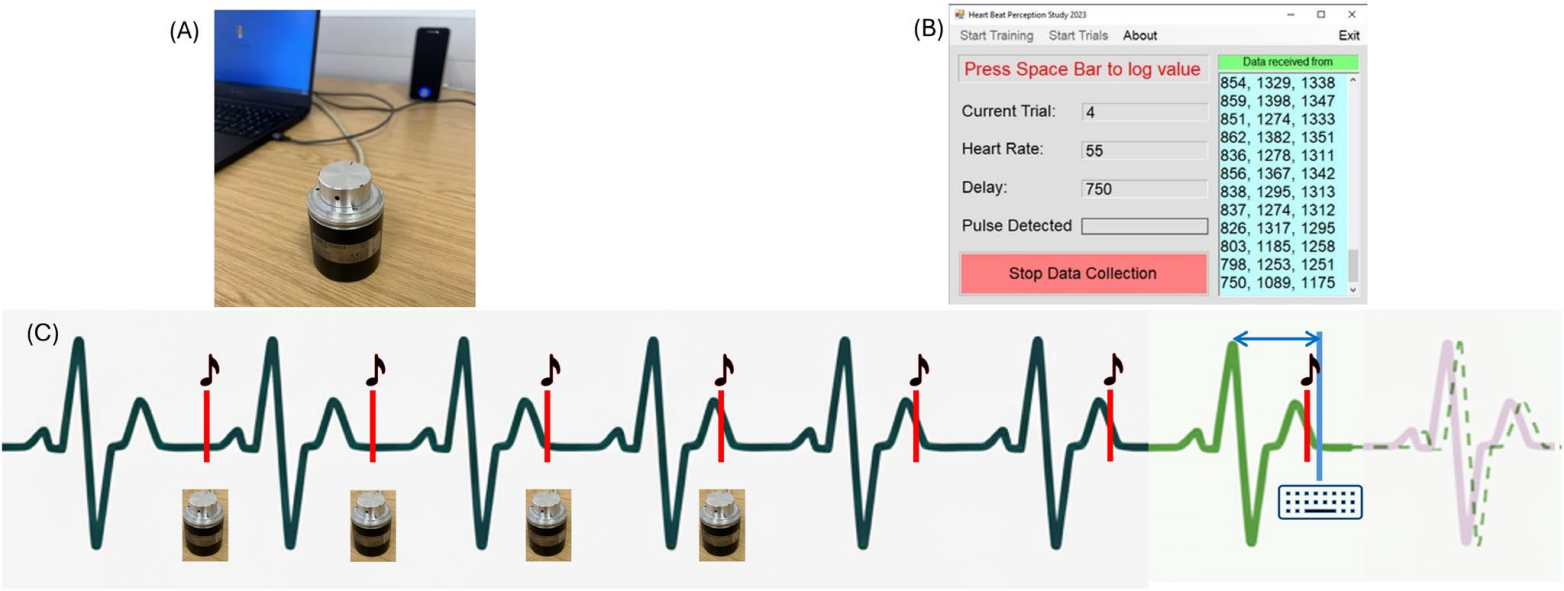
(Panel A) The 13‐bit optical Gray code encoder and dial that participants rotate with their dominant hand to adjust the delay between their R‐wave and the auditory tones played by the computer speakers. (Panel B) The software graphic user interface displayed to the experimenter. It displays in the main panel the trial number, live heart rate, live delay between R‐wave and tone based on the current position of the encoder, and in the right panel it shows the delay (first column), the observed period (second column), and the anticipated heart period (third column) over the preceding 12 cardiac cycles. (Panel C) Sample ECG recording illustrating how the ECG trace was used to compute and reference the key features of the task (i.e., tones, delays, observed heart period, and anticipated heart period) in the novel relative‐delay version of PAT‐ECG. Solid lines represent the recorded ECG signal. A tone sounded at the temporal position of each new R‐wave plus the delay set by the encoder. The position of a hypothetical delay choice is indicated by the vertical blue line and key‐press symbol. The delay corresponds to the gap (in milliseconds) between the most recent observed R‐wave (largest positive peak of the solid light‐green trace) and the point at which the spacebar was pressed (this interval is shown by the blue horizontal arrow). For analyses, the phase angle of the delay choice can be scaled relative to the observed heart period (light‐green R‐wave to purple R‐wave) and to the anticipated heart period (light‐green R‐wave to dotted‐line R‐wave). Red lines and the music‐note symbol indicate the timing of tones sounding once per cardiac period. The encoder image below the trace represents hypothetical dial movements that adjusted the position of the tone within the heart period. Typical trials (for perceivers) involve frequent dial adjustments at trial onset, followed by progressively smaller adjustments until the participant perceives synchrony between heartbeat and tone; at that point, dial movements temporarily cease, and once simultaneity is confirmed, the spacebar is pressed. The sequence shown here represents the end stages of a typical perceiver trial. The trace is not to scale.

The PAT presents participants with tones at their heart rate, but with variable delays relative to their heart beats (Plans et al. 2021). Before describing further details of the PAT‐ECG, it is important to note that there are, in principle, two alternate approaches to presenting tones in tasks that involve the perception of heartbeat‐tone synchrony: absolute delay or relative delay. When based on the absolute delay, an initial tone generated by the software at 100 ms after an R‐wave would, assuming no dial movements by the participant, reoccur at 100 ms after each successive R‐wave. However, because heart periods are naturally variable from one cardiac cycle to the next, basing the tones on absolute delay results in beat‐by‐beat changes in the phase of the cardiac cycle where the tone occurs (e.g., if 4 successive heart periods were 800, 900, 1000 and 1100 ms, the fixed R‐wave +100 ms tone position would mean the tone sounding at 45° of the first R‐R interval and only 33° of the last R‐R interval). Basing tones on the relative delay removes this variability by ensuring tones sound at the identical phase (assuming no dial movements) across successive heart periods. For instance, with the previous hypothetical sequence of heart periods, the tones would occur at R‐wave +100 ms, R‐wave +113 ms, R‐wave +125 ms, and R‐wave +138 ms to maintain the tone in the same relative phase (45°) of the lengthening cardiac cycles.

Existing heartbeat discrimination paradigms (e.g., Brener et al. 1993; Whitehead et al. 1977) and original implementations of the PAT (Plans et al. 2021) have relied on absolute‐delay timing during data capture. However, exploring relative‐delay task variants may help disentangle perceptual sensitivity to cardiac phase from assumptions about fixed post‐R‐wave latencies, and may offer a complementary perspective on cardiac interoception under conditions of natural heart‐rate variability. Recent evidence for phase‐based responding among some individuals (Palmer et al. 2025) make this development particularly important. Accordingly, as a second key feature of our implementation of the PAT, we introduce complementary PAT‐ECG versions, a novel version that presents tones using a relative delay during task performance and data capture, and a version that presents tones using absolute delays in line with existing PAT implementations.

The software we developed for the relative‐delay version of PAT‐ECG starts each trial by generating an audible 1000 Hz tone for 30 ms at a relative‐delay from the R‐wave corresponding with either 0/360°,^1^ 90°, 180° or 270° of the ongoing cardiac cycle.^2^ To permit relative‐delay scheduling during task performance, the beginning of the current cardiac cycle is marked by the most recent R‐wave, and its end is anticipated as the time of that R‐wave plus the mean duration of the three preceding measured heart periods (i.e., R‐R intervals). This anticipation allows tones to be scheduled in relative (phase‐based) time within the ongoing cardiac cycle during task execution. Our implementation applies a rolling three‐beat average throughout to anticipate the end of each ongoing (i.e., not completed) cardiac cycle. In this way the tones are presented not only at the same rate as the participant's beating heart (i.e., one tone per cardiac cycle) but also maintained in the same temporal location relative to the duration of the ongoing cardiac cycle across multiple cardiac cycles within each trial, despite the natural variability in the length of each cardiac cycle.

The dial can be rotated clockwise and counterclockwise and its angular range is mapped to coincide with the duration of the ongoing cardiac cycle to ensure that the full angle represents adjustments from 0° to 360° of the ongoing cardiac cycle duration. When participants rotate the dial, they alter the relative position of the tone. Due to the circular nature of this task, when the dial is rotated beyond its linear equivalent, the delay is set to the opposite end (i.e., when turning the dial clockwise beyond 360°, the tone is set to 0°, and vice versa).

As in the original task, participants are told that the PAT‐ECG tests how well they can synchronize the tones they hear with any heartbeats they might feel. They are instructed to rotate the dial with their dominant hand to adjust the delay between any heartbeats they can feel and the tone until they perceive the tones and their heartbeats to be synchronous (i.e., simultaneous). Participants are free to explore the stimuli over as many cardiac cycles as desired. They are instructed to press the spacebar on a standard computer keyboard with their non‐dominant hand to register their chosen delay, thereby ending the trial and silencing the tones. Participants are then free to self‐initiate the start of the next trial, whenever they feel ready, by pressing the spacebar.

The structure of a typical trial, and an image of the software graphic user interface that appears on a monitor visible to the experimenter, are shown in Figure 1B,C. The software writes to an output file at the end of each trial the chosen delay in milliseconds in relation to the most recently detected R‐wave. It also records the time to the subsequent R‐wave in order to calculate and write the precise duration of the heart period within which the space bar was pressed. Finally, it records a confidence score (how confident are you that you matched the tone to your heartbeat? 1 = not confident at all; 10 = extremely confident) that the participant is asked to provide after each trial.

For the alternative absolute‐delay implementation of the PAT‐ECG, tones are presented at absolute delays in milliseconds following each observed R‐wave and dial rotations adjust the delay in milliseconds. All other procedures are the same across the relative‐ and absolute‐delay task versions.

#### 1.2.2 Exteroceptive Task

In accord with the original PAT and earlier heartbeat detection tasks (e.g., Ring and Brener 1992; Plans et al. 2021), we also developed an accompanying exteroceptive task that can be used for participant familiarization prior to the PAT‐ECG, as a screener task before the PAT‐ECG, and/or as an exteroceptive comparison task, depending on the interests of the researcher. The exteroceptive task is designed to closely match the PAT‐ECG, but rather than synchronizing tones with their heartbeats, participants are asked to synchronize tones with a vibrotactile stimulus that is delivered by a 7‐cm diameter 26‐Ω Dayton Audio TT25‐16 circular vibrating tactor (actuator), weighing 250 g, affixed to the back of their non‐dominant hand (Figure 2). It is deemed exteroceptive rather than interoceptive because the stimulus is presented to the external surface of the skin, where it engages cutaneous somatosensory afferents rather than the visceral cardiac afferent pathways implicated in interoception (Desmedt et al. 2025; Khalsa et al. 2018; Vallbo et al. 1995). The tones and the vibrations are set to occur at a fixed frequency of 1 per second. The tone is initially set to play at 0°/360°, 90°, 180°, or 270° of the vibration‐to‐vibration intervals,^3^ with these start delays randomized across trials. Participants use the dial in exactly the same way as described in the PAT‐ECG above to adjust the delay between tone and vibration sensation and then use the space bar to lock in a chosen delay and end each trial. The Arduino acquisition software computer scripts and full details of how to replicate the hardware we have developed for the exteroceptive task are available here: https://github.com/Shaunmac68/PAT‐ECG.

**FIGURE 2:**
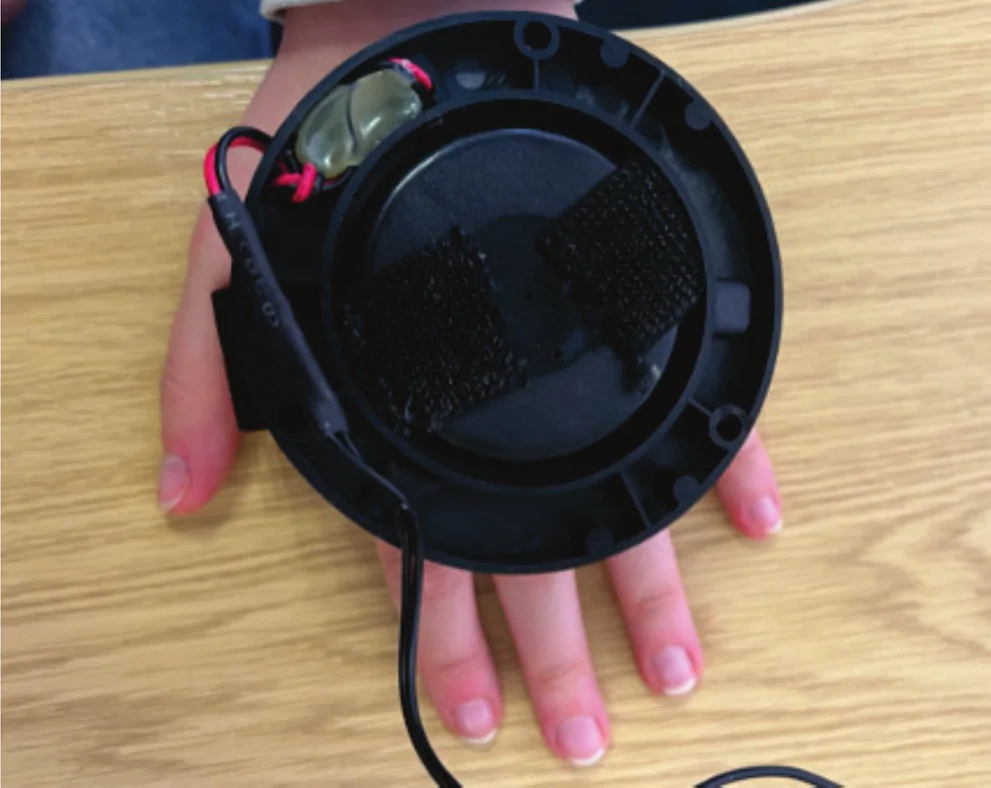
The 26‐Ω Dayton Audio TT25‐16 circular vibrating tactor that was affixed to the back of a participant's non‐dominant hand for the exteroception task.

#### 1.2.3 Analytic Approaches

Participants may perceive their heartbeat at different delays or phases, and thus there is no objectively “correct” response on any single trial of the PAT (Plans et al. 2021). Instead, interoceptive accuracy is inferred from the consistency of a participant's choices across trials. Various approaches can be used to quantify this consistency, all grounded in intertrial phase clustering (Lachaux et al. 1999). For the PAT‐ECG, we identified three parameters—each with two options—that define the algorithmic choices available.

The first parameter is mapping. As described in Palmer et al. (2026), consistency scores can be computed by mapping delays to their accompanying heart periods, which we refer to as variable‐mapping consistency, or by mapping to a single representative heart period, which we refer to as fixed‐mapping consistency.^4^

The second parameter is the heart period used for the calculation. The relative‐delay version of the PAT‐ECG allows permits both mapping approaches to be implemented using either the anticipated heart period (i.e., the value used online to schedule the tones during the task) or the observed heart period (i.e., the value extracted offline to represent the actual period during which the participant made their delay choice).

The final parameter is weighting by confidence. Under the weighted option, each trial's contribution to the mean resultant length is proportional to the confidence rating. Under the unweighted option, each chosen delay contributes equally. This 2 (mapping) × 2 (heart period) × 2 (confidence weighting) structure yields eight possible analytic approaches. These are examined in the following sections, with key terminology summarized in Table 1.

**TABLE 1:** Glossary of key terms.

Relative delay	A relative delay remains in the same relative position within each cardiac cycle, despite any beat‐by‐beat changes in heart period (e.g., if 4 successive heart periods were 800, 900, 1000, and 1100 ms and the initial tone sounded at R‐wave +100 ms, the subsequent tones would, assuming no dial movements, occur at R‐wave +113 ms, R‐wave +125 ms, and R‐wave +138 ms to maintain the tone in the same relative phase of the lengthening cardiac cycles). This may also be referred to as phase delay (Palmer et al. 2026) or angular delay since it defines the delay in angular space (degrees). The PAT‐ECG implementation used in the current study presents tones using relative delays
Absolute delay	An absolute delay remains in the same absolute position within each cardiac cycle, despite any beat‐by‐beat changes in heart period (e.g., if 4 successive heart periods were 800, 900, 1000, and 1100 ms and the initial tone sounded at R‐wave +100 ms, subsequent tones would, assuming no dial movements, remain at R‐wave +100 ms to maintain the tone in the same absolute position despite the lengthening cardiac cycles). This may also be referred to as fixed‐period delay (Palmer et al. 2026) since it defines the delay in absolute time (milliseconds). Previous PAT implementations (Plans et al. 2021; Palmer et al. 2026) presented tones using absolute delays
Anticipated heart period	The duration between the most recently observed R‐wave, and the anticipated occurrence of the next R‐wave, calculated as the mean duration of the three preceding heart periods. This method of measuring each R‐wave and anticipating the occurrence of the next one to map the predicted duration of an ongoing cardiac period to a full cycle during task execution is applied in the relative‐delay PAT‐ECG to scale the encoder on a beat‐by‐beat basis. Previous PAT implementations also used recently observed heart periods to dynamically adjust the scaling of their virtual dial (Palmer et al. 2026). In contrast, in the present implementation this rolling average is additionally used to ensure tone presentations represent relative delays, that is they are in relative (phase angles) rather than absolute (millisecond) terms across cardiac cycles during task performance
Observed heart period	The duration between the most recently observed R‐wave prior to the space bar press on a PAT‐ECG trial and the next observed R‐wave. This measure is extracted offline. A comparison of the observed and the final anticipated heart period for each trial can give an indication of the accuracy of the anticipated heart period algorithm. In the PAT‐ECG implementation used in the current study, tones were scaled in relative time using the anticipated heart period during task execution. Delay choices can however be rescaled offline to the observed heart period for analyses
Variable‐mapping consistency	This approach to calculating consistency aligns with the relative delay conceptualisation of heartbeat perception. It assumes that individuals who can accurately perceive their heartbeats will do so in the same phase of their cardiac cycle across trials. Accordingly, the mapping between a full angle (360°) and time constituting a full cycle varies from trial to trial to ensure that each heart period maps on to a full cardiac cycle. For example, a chosen delay of 240 ms (on a trial with a heart period of 1200 ms) and 140 ms (on a trial with a heart period of 700 ms) represents the same phase—the preferred delay is in the same relative position (i.e., at 72°, or 1.26 rad) of the heart period. This may also be referred to as phase‐based consistency (Palmer et al. 2026). Although this analytic approach is most naturally paired with data collected using relative delays, it can also be applied retrospectively to data collected using absolute delays (e.g., Palmer et al. 2025)
Fixed‐mapping consistency	This approach to calculating consistency aligns with the absolute delay conceptualisation of heartbeat perception. It assumes that individuals who can accurately perceive their heartbeats will do so at a similar absolute delay from the onset of the cardiac contraction marked by the R‐wave (or more importantly, relative to the opening of the aorta and the ejection of blood from the left ventricle) across trials regardless of the duration of the cardiac cycles. Accordingly, the mapping between a full angle (360°) and time constituting a full cycle does not vary from trial to trial and is instead fixed to a single value, representative of a large pool of periods. This may also be referred to as delay‐based consistency (Palmer et al. 2026). Although this analytic approach is most naturally paired with data collected using absolute delays, it can also be applied retrospectively to data collected using relative delays

##### 1.2.3.1 Variable‐Mapping Consistency (Palmer et al. 2026)

This approach assumes that individuals who can accurately perceive their heartbeats will do so in the same phase of their cardiac cycle across trials. Accordingly, the mapping between a full angle (360°) and time constituting a full cycle varies from trial to trial to ensure that each heart period maps on to a full cardiac cycle. Meanwhile, the delays chosen by non‐interoceptive participants would be expected to distribute more uniformly (i.e., more random spread) around the cardiac cycle across trials. To assess consistency across trials, each chosen delay is transformed into an angle describing its position relative to the duration of their heart period for the corresponding trial.^5^ An individual's score of consistency across trials is the mean resultant length of unit‐length vectors with angles obtained by multiplying the ratio *d*/*p* (where *d* = chosen delay and *p* = period) by 360 for degrees or by 2*π* for radians. The mean resultant length is computed as

$$n{\left(R\right)}^{-1}\left|\sum_{r\in R}e^{i\theta_{r}}\right|$$

where $e^{i\theta_{r}}$ refers to a unit‐length vector of angle $\theta_{r}$, with *i* indicating the imaginary operator, *r* representing any trial belonging to the set of all trials *R* from the same recording session (or condition), and *n*(*R*) indicating the number of trials. The mean resultant length can be equivalently computed by expanding Euler complex‐valued vector notation into the sine and cosine vector projections

$$\sqrt{{\left[n{\left(R\right)}^{-1}\sum_{r\in R}\cos\left(\theta_{r}\right)\right]}^{2}+{\left[n{\left(R\right)}^{-1}\sum_{r\in R}\sin\left(\theta_{r}\right)\right]}^{2}}$$

The mean resultant length reflects consistency with a higher value (max 1) indicating greater clustering of the angles towards the same direction (i.e., greater consistency), and a lower value (min 0) indicating a more uniform spread of the angles (i.e., lower consistency) across trials.

##### 1.2.3.2 Fixed‐Mapping Consistency (Palmer et al. 2026)

This approach assumes that individuals who can accurately perceive their heartbeats will do so at a similar absolute delay from the R‐wave, which provides a distinct temporal event that reliably precedes mechanical cardiac contraction, across trials, regardless of the duration of the cardiac cycles. Accordingly, the mapping between a full angle (360°) and time constituting a full cycle does not vary from trial to trial and is instead fixed to a single value, representative of a large pool of periods. For example, this could be the median of the observed (anticipated) heart period across trials for a participant to ensure individual representability (e.g., Palmer et al. 2026). This approach is supported by the notion that heartbeat perception is constrained by pulse‐arrival time (i.e., interval between R‐wave and the arrival of the resulting blood pressure pulse at a peripheral site such as the wrist), which typically falls within a 100–300 ms window and shows limited variability across the range of heart periods typically observed in resting adults (approximately 600–1200 ms) (Brener et al. 1993). In this case, consistent heartbeat perception may reflect the detection of mechanoreceptive feedback occurring at a similar delay after each cardiac contraction. To assess consistency, each chosen delay is transformed into an angle relative to a fixed representative heart period, regardless of the duration of the heart period across trials (Palmer et al. 2026). Cross‐trial consistency is then computed using the mean resultant length formula described in the previous section. The demonstration section below provides some examples of how the consistency scores can be used in research and practice.

##### 1.2.3.3 Confidence‐Weighted Consistency

In addition to computing consistency scores via standard formulation (i.e., unweighted; all trials contribute equally to the mean resultant length), we also introduced a novel method whereby we calculated confidence weighted versions for each approach. Specifically, a greater weight was attributed to the data from trials where the participant reported greater confidence in their choice, relative to other trials. This was done on the basis that on trials where participants are relatively lower in confidence, they may have been simply guessing, hence those trials may contain some random noise that is absent from trials where the perception was certain. Importantly, this algorithm weighs trials by the within‐participant variation in confidence and is not influenced by the average confidence reported by the individual. Confidence‐weighted consistency was computed as

$$\frac{\left|\sum_{r\in R}w_{r}e^{i\theta_{r}}\right|}{\sum_{r\in R}w_{r}}$$

where the numerator computes the resultant length of non‐unit‐length vectors of angle $\theta_{r}$ and length $w_{r}$, with the former defining the choice and the latter the confidence in the choice. The denominator normalizes the weighted metric to the 0–1 range.

##### 1.2.3.4 Exteroceptive Task

Consistency across trials on the exteroceptive task is calculated using the identical formulas as provided in the previous sections, with the period being 1000 ms as determined by the pre‐programmed intervals between the exteroceptive vibrations.

### 1.3 Task Demonstration

Having described the PAT‐ECG and accompanying exteroceptive task, we include a demonstration dataset (*N* = 91) to show the types of data the tasks generate and how they can be analyzed.

#### 1.3.1 Participants

We collected data from 91 consenting adult volunteers (49 male, 42 female, *M*_age_ = 20.7, SD = 3.1). They were instructed to refrain from consumption of alcohol and medication in the 6 h before testing and to refrain from consuming any caffeine 2 h prior to the experiment. These restrictions were implemented to minimize acute influences on cardiovascular and autonomic functioning, including physiological signals relevant to cardiac perception (e.g., Leganes‐Fonteneau et al. 2025), and are consistent with recommended practice in cardiovascular psychophysiological research (Quigley et al. 2024). Participants reported no present illness and no known neurological disorders or heart defects. We decided a priori to exclude participants who breached any of these criteria because known cardiac defects (e.g., arrhythmias) may affect the generation and perception of cardiac signals, whereas current illness and neurological disorders may influence physiological and cognitive functioning, potentially increasing variability in task performance (e.g., Hallett et al. 2022). The study protocol received a favorable opinion from our local research ethics committee.

#### 1.3.2 Design

The experiment adopted a within‐person design. Participants completed 8 familiarization and 28 experimental trials of our novel relative‐delay version of the PAT‐ECG to provide initial empirical data on this relative‐delay approach to cardiac interoceptive accuracy assessment. They also did 8 practice and 28 experimental trials of the accompanying exteroceptive task. We decided on 28 trials based on modeling using simulated PAT data (Plans et al. 2021; Palmer et al. 2026) demonstrating that reliable estimates of cardiac interoceptive accuracy can be generated from as few as 15 trials and the benefit of additional trials was very modest beyond 20. Our choice of 28 trials thereby represented a good balance between comfortable task duration for participants and the number of observations required to make reliable classifications.

#### 1.3.3 Procedure

Participants were scheduled to attend a single 90‐min appointment to complete their participation. They were welcomed to the laboratory, briefed, and provided informed consent. Participants were then seated at a desk, instrumented for the exteroceptive task, and provided with exteroceptive task instructions. They subsequently completed 8 familiarization and 28 experimental trials of the exteroceptive task. They were then instrumented for ECG recording. We used pre‐gelled spot electrodes (BlueSensor SP, Ambu, Cambridgeshire, UK) placed on the right clavicle and lowest left rib, with a ground electrode on the left clavicle. The ECG signal was amplified (Bagnoli‐4, Delsys, Boston, MA), filtered (1–100 Hz), and digitized at 2500 Hz with 16‐bit resolution (CED Power 1401, Cambridge Electronic Design, Cambridge, UK) using Spike2 software (Cambridge Electronic Design). Participants were then provided with PAT‐ECG instructions. Importantly, to reduce the risk of participants adopting strategies such as holding their breath or tensing their muscles in an attempt to amplify heartbeat sensations, the instructions specifically asked participants to sit as still as possible, breathe normally, and refrain from tensing their muscles throughout the task. After instruction, they completed 8 familiarization and 28 experimental trials of the relative‐delay PAT‐ECG, whereas the experimenter visually monitored participants and noted no obvious breaches of the task instructions. At the end of each PAT‐ECG trial, participants were asked to rate their confidence in matching the tone to their heartbeat on a scale from 1 (not confident at all) to 10 (extremely confident). The 28 trials of the exteroceptive task took approximately 10 min, and the 28 trials of the PAT task took approximately 30 min to complete. On completion of all tasks, the recording equipment was removed, and participants were thanked and debriefed. Allowing time for briefing, instrumentation, completion of both tasks, de‐instrumentation, and debriefing, total participation time ranged from approximately 60–90 min.

#### 1.3.4 Data Preprocessing

The first step of our data analysis pipeline involved removing bad trials. Any experiments involving ECG recording are susceptible to occasional bad trials due to the following principal reasons: (a) confounding events in the environment (e.g., surprising external noises that may cause distraction mid‐trial); (b) ECG signal artifact (e.g., occurring due to participant movement).

We mounted two lines of defense to ensure no bad trials were included in our analyses. First, the experimenter monitored the signals and the laboratory environment throughout all testing sessions. Whenever he detected potentially confounding events (e.g., door closing in adjacent lab) or was alerted to their presence by the participant (e.g., “I pressed space bar by accident and ended the last trial prematurely”), and whenever he observed signal artifact, he made a note to reject that trial, and he added a replacement trial to the protocol.^6^ The first line of defense ensured that all participants completed 28 trials that were initially deemed as suitable for inclusion by the experimenter.

Second, we performed offline removal of any trials with a heart period (or anticipated heart period) that was greater than 5 robust standard deviation units from the median heart period (or anticipated heart period) across trials for that participant. As a measure of robust standard deviation, we used median absolute deviation (MAD) multiplied by 1.4826 because it is less skewed by outliers and less sensitive to non‐normality than the conventional standard deviation (Leys et al. 2013). We settled on 5 units of the robust standard deviation from the median as the cutoff based on modeling with parameters ranging from 2 to 10 MAD units revealing that in our dataset 5 robust standard deviation units achieved the best balance of rejecting obviously outlying heart periods without rejecting any periods that were deemed biologically plausible based on the surrounding periods for each participant. This second line of defense identified 6 (observed heart period), 4 (anticipated heart period), and 1 (observed and anticipated heart period) outlying trials (i.e., < 1% of total trials) for removal.

#### 1.3.5 Data Reduction and Analysis

##### 1.3.5.1 Participant Screening and Exclusion

After removing noisy ECG data, we proceeded to the screening and potential removal of participants. Specifically, in line with previous experiments (e.g., Plans et al. 2021), we decided to exclude any participants who performed poorly on the initial exteroceptive screening task. Given the strong and obvious exteroceptive stimuli that we used in that task, the most likely reasons for being classed as non‐exteroceptive on our exteroception task would be poor comprehension of the task, low motivation and/or attention, or inability to synchronize signals (Plans et al. 2021).

We analyzed performance (i.e., consistency indexed by the mean resultant length described in Section 2.2.3) on the exteroceptive task using a Monte Carlo simulation of phase delays to define a resultant score 95th percentile threshold for classification (Palmer et al. 2026). To calculate the threshold for classification we simulated 28 trials with a fixed 1000 ms period to match the parameters of the exteroceptive task, we simulated delays based on a uniform distribution across the full 1000 ms range, and we calculated the mean resultant length. The process was repeated 100,000 times, and then the 95th percentile of the resultant values was computed and used as the classification threshold. The analyses revealed that 84 (92%) participants scored beyond the *R* = 0.33 threshold and were thereby classified as exteroceptive. The 7 non‐exteroceptive participants were removed from the dataset ahead of PAT‐ECG analyses on the basis that they likely had poor task comprehension, poor engagement, or general perception difficulties that could have distorted their performance.

##### 1.3.5.2 PAT‐ECG Classification Analyses

A key utility of the PAT is its ability to classify participants as interoceptive or non‐interoceptive based on the consistency of their delay choices.^7^ Approximately 17% of participants were classified as interoceptive using the absolute‐delay PPG implementation of the PAT during a single PAT assessment at rest (Palmer et al. 2026). To allow comparison, we performed a series of Monte Carlo simulations of phase delays to define consistency scores corresponding to the 95th percentile threshold for classification (Palmer et al. 2026). As per Palmer et al. (2026), participants were classified based on two types of consistency: variable‐mapping consistency and fixed‐mapping consistency, as described below. For each type, we computed four versions of the consistency score, crossing two factors: (1) whether heart period data were anticipated or observed, and (2) whether trials were weighted by their associated confidence rating or treated as unweighted.

##### 1.3.5.3 Classification: Variable‐Mapping Consistency

To calculate the threshold for classification via variable‐mapping consistency, we simulated the number of valid trials per participant trials using their observed single heart periods (*p*) (and anticipated heart periods) and delays uniformly drawn from 0 to *p* to cover the full range of delay possibilities. We calculated the phase angles and mean resultant length. The process was repeated 100,000 times, and then the 95th percentile of the resultant values was used as the classification threshold—if the observed scores exceeded the threshold the participant was deemed as interoceptive due to showing a degree of phase consistency that is highly unlikely (< 5% probability) to have arisen by chance. This procedure is similar to that adopted by Palmer et al. (2026) with the following key exceptions. They: (a) simulated heartbeat periods from 500 to 1500 ms for all participants whereas we used the recorded periods for each participant; (b) drew delays at random from ± ½ *p* whereas we used the full range of possibilities for each participant; and (c) simulated a fixed‐number of trials (i.e., 17, to match the minimum valid trial number across their participants). Their approach thereby generated a single classification threshold for all participants. Our approach alternatively generates individual classification thresholds for each participant based on simulations of random responding from within the parameters of their observed data.^8^

##### 1.3.5.4 Classification: Fixed‐Mapping Consistency

To calculate the threshold for classification via fixed‐mapping consistency, we used the same broad approach except here the simulation used a constant heart period (Palmer et al. 2026). Specifically, we computed the median of observed (anticipated) heart period across all trials per participant in order to specify an individualized fixed‐heart period per participant based on their data. On occasions where delay choices exceeded an individual's median heart period, that trial was removed. This step was undertaken because those chosen delays would be represented by angles greater than the full angle and, therefore, would be misrepresenting the phase of the cardiac cycle. This was a rare occurrence impacting only 1.6% of total trials; all participants retained at least 22 valid trials, with the majority retaining all 28 trials. The median scaling is based on the approach of Palmer et al. (2026).

For each participant, 100,000 simulations were run using their fixed period to generate an individualized null distribution of resultant lengths. The 95th percentile of this distribution served as that participant's individual classification threshold. Observed values exceeding this threshold indicated greater‐than‐chance consistency, and the participant was classified as interoceptive (Palmer et al. 2026). The code for these analyses is provided in Supporting Information.

##### 1.3.5.5 Heart Period Analyses

Aside from our classification analyses, we also tested for any differences between the final anticipated heart period that was calculated during task performance and the observed heart period that was extracted offline. This analysis can provide some insight into the performance of the anticipated heart period algorithm.

## 2 Results

Our results are presented to support understanding of the task and to guide readers in developing their own research questions, applications, and analytic strategies where the PAT‐ECG may be useful. They are not intended to provide population‐level estimates of the prevalence of heartbeat perception.

### 2.1 Comparison of Observed Versus Anticipated Heart Period

We computed the median of the differences between the final anticipated heart period and the observed heart period on each trial and conducted a one‐sample *t*‐test across participants to determine whether these differences were, on average, significantly greater than zero (Figure 3). The analysis revealed a significant effect, indicating that the anticipated heart period (based on the average of the most recent three cardiac cycles) tended to be longer than the observed heart period. Although this bias was systematic and statistically significant, it was small in biological terms (median difference < 5 ms).

**FIGURE 3:**
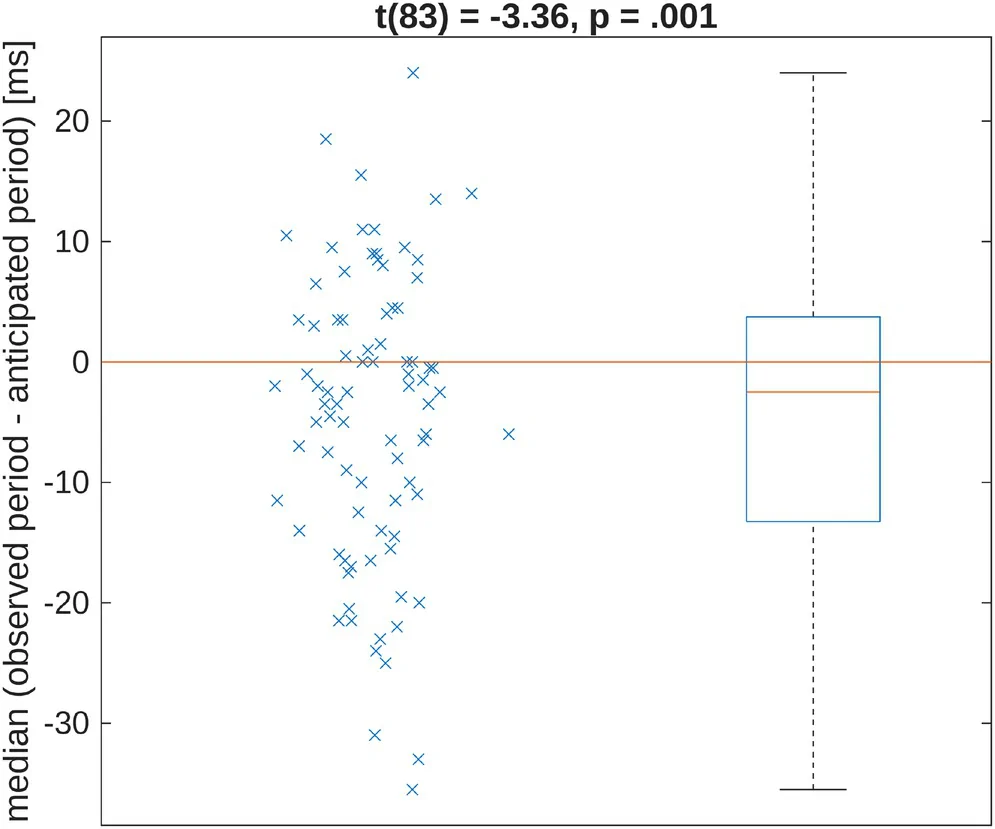
Median values of the heart period change from the average of the three cycles before the spacebar press (anticipated heart period) to the cycle wherein participants pressed the bar (observed heart period) values. The observed heart period that contained the synchrony decision and bar press tended to be shorter (i.e., faster heart rate) than the anticipated heart period.

### 2.2 Classification Analyses

Fourteen participants (16.7%) were classified as interoceptive via at least one of the eight classification routes. Their mean resultant lengths are plotted in Figure 4. In brief, ten were classified as interoceptive across all eight analysis types (11.9%; participants 1, 2, 3, 7, 8, 9, 10, 11, 13, 14 in Figure 4). The others were classified as interoceptive via seven (participant 12), six (participant 4), five (participant 5), and four (i.e., all their confidence weighted analyses—participant 6) out of the eight analysis types. Figure 4 indicates which specific analyses yielded interoceptive versus non‐interoceptive classifications for these participants.

**FIGURE 4:**
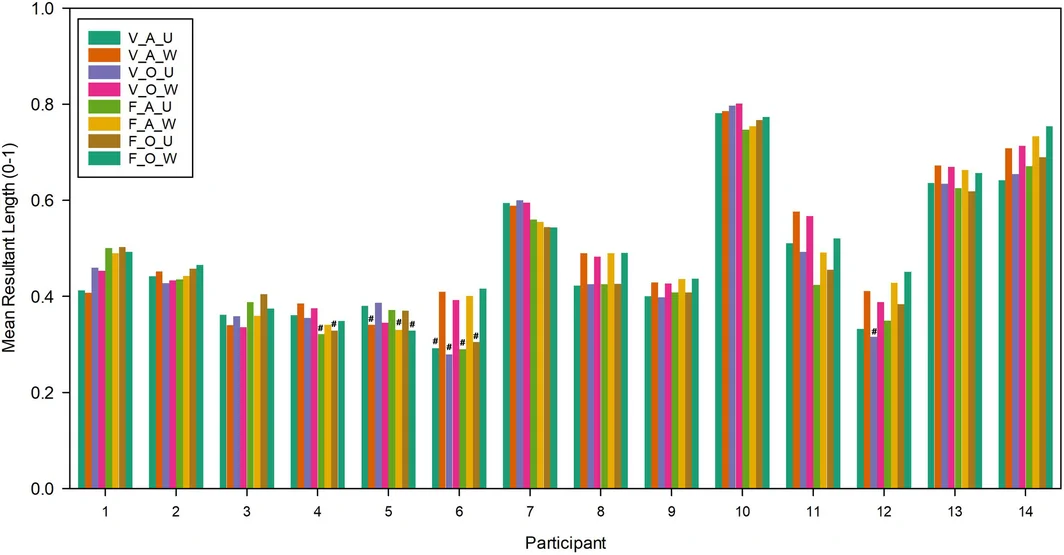
Mean resultant length (0–1) for the 14 participants classed as interoceptive via at least one of the eight classification approaches. AE, anticipated heart period; F, fixed‐mapping consistency; O, observed heart period; U, unweighted; V, variable‐mapping consistency; W, weighted. ^#^Indicates any mean resultant lengths that were classed as non‐interoceptive.

To permit comparison with Palmer et al. (2025), who compared classification rates for unweighted observed variants of the fixed‐mapping and variable‐mapping consistency approaches, we note that 13 participants in our sample (15.5%) were classified as interoceptive by at least one of these methods. Palmer et al. (2025) classified 17.0% of their screened sample as interoceptive via at least one of these methods using their absolute‐delay PAT implementation (i.e., *interoceptive combined*). Importantly, eleven of our interoceptive participants (13.1%) were classified as interoceptive by both fixed‐mapping and variable‐mapping consistency approaches (referred to as *interoceptive ambiguous* by Palmer et al. 2025; represented 9.6% of their screened sample), whereas one participant (1.2%) was classified only via fixed‐mapping consistency (referred to as *delay‐interoceptive* by Palmer et al. 2025; 3.5% of their screened sample), and one participant (1.2%) was classified only via variable‐mapping consistency (referred to as *phase‐interoceptive* by Palmer et al. (2025); 3.9% of their screened sample). Two‐proportion *z*‐tests comparing Palmer et al.'s (2025) classification rates with ours revealed no significant differences (interoceptive combined, *z* = −0.18, *p* = 0.86; interoceptive ambiguous, *z* = 0.93, *p* = 0.35; delay‐interoceptive, *z* = −1.11, *p* = 0.27; phase‐interoceptive, *z* = −1.22, *p* = 0.22). The summary dataset reporting all individual participant data are available here in Supporting Information.

## 3 Discussion

The PAT‐ECG is designed to overcome the key limitations of heartbeat counting and heartbeat detection cardiac interoception tests and build on the original PPG version of the PAT to provide a rigorous assessment of cardiac interoceptive accuracy. This paper has outlined the new implementation and provides resources to facilitate its use. It also provides a demonstration of the relative‐delay PAT‐ECG and accompanying exploration of various approaches to scale data, apply weightings to the data, and classify participants as interoceptive or non‐interoceptive.

Our data revealed 16.7% of participants to be classified as interoceptive via at least one of our analytic approaches, based on their performance on the PAT‐ECG at rest. This does not differ significantly from the 17% of participants who were classified as interoceptive by at least one of the unweighted variable‐mapping or fixed‐mapping equivalents reported by Palmer et al. (2025) in their analysis of data from the absolute‐delay PPG implementation of the PAT. These comparisons are presented descriptively to illustrate broadly similar classification proportions across implementations under supervised laboratory conditions and should not be interpreted as evidence of validity or equivalence between implementations. Rather, the development of PAT‐ECG provides a foundation for future studies that can directly compare ECG and PPG implementations.

By presenting 2 mapping (variable, fixed) × 2 heart period (anticipated, observed) × 2 weighting (unweighted, confidence‐weighted) combinations of PAT‐ECG analytic options, our data also demonstrate the analysis routes that can be taken by future users.

### 3.1 Effects of Heart Period

A first choice for future users of the relative‐delay PAT‐ECG is whether to retain scaling of delay choices to the anticipated heart period that was used to schedule tones during task performance or rescale offline to the observed heart period. Importantly, our data indicate limited empirical consequence of this decision. The difference between the anticipated and observed heart periods was biologically very small (< 5 ms), and the scaling choice only impacted classifications for two participants (participants 5 and 12; difference between mean resultant lengths calculated using observed versus anticipated heart periods < 0.02). The different classifications for these two participants reflect their mean resultant scores being right on the borderline of their classification thresholds, and not significant task‐performance differences between the two different heart periods. Therefore, if classifying participants as interoceptive or non‐interoceptive via the relative‐delay PAT is a primary experimental aim, either the default scaling to the anticipated heart period (using the current estimation algorithm) or offline scaling to the observed heart period appears sufficient. Users of absolute‐delay PAT variants (i.e., existing PPG implementations and the absolute‐delay PAT‐ECG) face no comparable timing decision, as scheduling absolute‐delay tones depends only on observed periods.

### 3.2 Effects of Confidence Weighting

The decision to adjust trial‐weighing based on confidence ratings or weigh trials evenly also had relatively little impact on the classification outcomes of the current sample. The rationale for using weighted resultant scores stems from the intuitive notion that researchers interested in heartbeat perception would do well to place greater emphasis on trials where participants rate greater certainty in their perception, compared with trials in which they may have been guessing. On this basis, one could tentatively hypothesize that more participants would be classed as interoceptive using weighted than unweighted scores. In reality, one participant was classed as interoceptive in the weighted analysis but not in the unweighted analyses (Figure 4, participant 6). Equally, another participant fared better with unweighted scores (Figure 4, participant 5). The modest impact of confidence weighting may reflect our observation that within‐person variability in confidence ratings across trials was relatively small (*M* within‐person confidence rating SD across trials = 0.91, between‐person SD of *M* confidence ratings = 2.02), corresponding to an approximate intraclass correlation coefficient of 0.83. In other words, over 80% of the variance in confidence ratings was due to stable differences between participants, with relatively little variance from trial‐to‐trial fluctuations within a participant.

Future studies could explore alternate approaches to confidence assessment (see for example Palmer et al. 2026), examine the impact of different aspects of confidence (e.g., confidence in feeling heartbeat versus confidence in matching the tone), or manipulate features that may impact heartbeat perception (e.g., exercise, stimulant drugs) across multiple PAT‐ECG assessments to determine whether greater divergence between weighted and unweighted scores emerges. Although it had little impact on classification rates, there was anecdotal evidence of longer mean resultant lengths for confidence weighed scores for some of our interoceptive participants (e.g., Figure 4, participants 6, 8, 9, 11, 12, 13, 14), providing some encouragement for future examination of this factor. Alternatively, one can make a case for always using unweighted scores on the basis that interoception is a multidimensional construct and the confidence ratings reflect one's metacognition regarding their interoceptive decisions, which should not be conflated with objective interoceptive accuracy as is assessed via PAT‐ECG (Garfinkel et al. 2015).

### 3.3 Effects of Variable‐ and Fixed‐Mapping Consistency

Although decisions concerning the heart period and whether to weight the analyses on confidence ratings have little impact on PAT‐ECG classification, there appears to be value in using both variable‐mapping and fixed‐mapping consistency analyses. This is because there are physiological rationales and empirical evidence to support the idea that genuine heartbeat perceivers can emerge uniquely from one method or the other (Palmer et al. 2025). For instance, one of the physiological explanations of heartbeat perception contends that perception occurs when individuals detect a mechanical event associated with the arrival of a blood pressure pulse at a part of their body, and that the pulse arrival time at a particular bodily location is generally consistent across cardiac cycles (with the delay depending on the location—Ring and Brener 1992). This conceptualization of heartbeat perception is captured by a fixed‐mapping consistency approach to compute PAT resultant scores, where the data are scaled to a fixed circumference to ensure that the preferred absolute delay occurs at the same angle across trials, regardless of heart period variability (Palmer et al. 2026).

An alternative physiological explanation is that heartbeat perception occurs in a particular phase of the cardiac cycle, and the position of this phase is not governed by a fixed absolute delay; instead, the preferred delay adjusts proportionally to any beat‐by‐beat lengthening or shortening of the cardiac cycle. For instance, it has been speculated that for some individuals, perception occurs not in relation to pulse arrival, but to vascular distension offset (e.g., the release of the pulse pressure wave) or vessel recoil—events that may occur more consistently in phase angle rather than in absolute time after an R‐wave (McDonald 1955; Palmer et al. 2025). This conceptualization of heartbeat perception is captured by a variable‐mapping consistency approach to compute PAT resultant scores to ensure delay choices are expressed relatively to the changing heart periods over trials (Palmer et al. 2025).

Our data revealed that the majority of participants who were classified as interoceptive emerged from both the fixed‐mapping and the variable‐mapping consistency methods. Palmer et al. (2025) who introduced this approach made the same observation with data obtained from the PPG implementation of PAT. This may reflect the data being collected at seated rest, with relatively consistent heart periods across trials. When heart periods are relatively stable across trials, the two methods will naturally converge. However, we did find evidence for two participants who, based on their unweighted data, were classified as interoceptive by the variable‐mapping consistency (Figure 4, participant 4) and the fixed‐mapping consistency (Figure 4, participant 12) methods only. This adds to evidence reported by Palmer et al. (2025) for a minority of interoceptive participants emerging uniquely from variable‐mapping and fixed‐mapping approaches, endorsing the use of both analytic options.

### 3.4 Strengths, Limitations, and Future Directions

Although both fixed‐mapping and variable‐mapping consistency analyses can be applied to data collected using either absolute‐delay (Palmer et al. 2025) or relative‐delay (current study) PAT variants, these analytic approaches are mathematically optimized when paired with their corresponding data‐collection method. Accordingly, to maximize sensitivity to both delay‐ and phase‐based responding, future studies could consider administering both PAT‐ECG variants to the same participants, applying fixed‐mapping consistency analyses to data from the absolute‐delay PAT‐ECG and variable‐mapping consistency analyses to data from the relative‐delay PAT‐ECG. This consideration may be particularly important in conditions characterized by substantial inter‐trial variability in heart period (e.g., during recovery from exercise), where the method used to present tones during data collection (e.g., absolute‐delay scheduling) may adversely affect the sensitivity of the non‐partnered analytic approach (e.g., variable‐mapping consistency). As the PAT‐ECG allows straightforward implementation of both variants, future research could therefore systematically examine their relative utility across different experimental contexts (e.g., rest, exercise, and recovery).

Although administering two PAT variants may be advantageous in some settings, it is also worth noting that when heart periods are relatively stable across trials, the choice between fixed absolute‐ or relative‐delay scheduling during task execution is unlikely to substantially compromise the ability of a non‐partnered analytic method (i.e., variable‐ versus fixed‐mapping consistency) to detect interoceptive responding. Under such conditions, a single assessment using either the absolute‐ or relative‐delay PAT variant is likely to be sufficient to identify individuals performing above chance, provided that both fixed‐mapping and variable‐mapping analyses are applied offline (Palmer et al. 2025).

We concede that our exteroceptive task used regular intervals between vibrations and tones and was therefore not a perfect match to the PAT‐ECG where the intervals between heartbeats and tones naturally vary. Ultimately the choice of fixed vs. variable intervals depends on the aims of including the exteroceptive task. For familiarization the use of the same fixed interval for all participants may be acceptable. For general screening for attention and/or motivation, it may be preferable to have variable intervals that approximate those expected during the PAT‐ECG. To fully match task difficulty at the individual level, it would be necessary to use the exact intervals from the PAT‐ECG. Although we have not used all variants in this project, future studies can adjust our script to match for various purposes, including matching intervals on an individual participant level as has been used in previous exteroceptive tasks designed for use with the PAT (e.g., Plans et al. 2021).

Given that the principles underpinning the PAT are based on consistency, future studies would do well to instruct participants to focus on a single location (e.g., chest) when scanning for their heartbeats, and/or measure the location of their heartbeat perception trial‐by‐trial (Palmer et al. 2026). Different locations used to make heartbeat judgments may have different pulse arrival times and preferred delays and hence any inconsistency in locations used across trials would introduce noise to the data and may underestimate interoceptive participants (Ring and Brener 1992). Future studies could also incorporate respiratory belts and electromyographic sensors to provide a more objective assessment of participant compliance with instructions to remain still, breathe normally, and avoid muscle tension during trials.

We encourage future research to further scrutinize the PAT‐ECG via direct comparison with other PAT implementations and other cardiac interoceptive accuracy tests, and via computation of test–retest and/or split‐half reliability, where trial numbers permit. Future studies can also examine the validity and sensitivity of our method via assessment during manipulations thought to alter heartbeat perception (e.g., exercise, stimulant drugs) (Khalsa et al. 2009; Montgomery et al. 1984) and/or by comparing control populations with populations associated with atypical interoception. Repeated PAT‐ECG assessments across multiple conditions (e.g., rest, exercise, and recovery) and timepoints are especially recommended (Plans et al. 2021; Murphy 2024). For example, such an approach would enable researchers to identify specific contexts in which heartbeat perception is more likely to emerge, whereas distinguishing between individuals who consistently, intermittently (e.g., only under certain conditions), or never exceed the classification criterion. This distinction is important because a single resting assessment cannot distinguish individuals who never perceive their heartbeat signal from those who may perceive it under specific conditions (Wittkamp et al. 2018).

Finally, while our demonstration adopted a classification‐based approach to facilitate comparison with previous PAT research, it should be noted that PAT‐ECG scores may also be analyzed as continuous measures. The choice between continuous and dichotomous approaches has both benefits and trade‐offs and thus should ultimately be guided by the research question and aims. Comprehensive evaluation of the relative merits of these approaches was not a focus of the present study, and interested readers are referred to Palmer et al. (2026) for a more detailed discussion.

## 4 Conclusion

We have presented a new ECG‐based implementation of the PAT that addresses potential limitations of existing interoceptive accuracy assessments. All resources have been made openly available, and we provide demonstration data to facilitate adoption and further development of the method. We encourage researchers to adopt, evaluate, and extend this approach. The original PAT implementations (Plans et al. 2021; Palmer et al. 2026) established an important advancement in interoceptive accuracy assessment, and we propose that the present ECG‐based extensions further strengthen this methodology and support future applications that will advance the interoception field.

## Author Contributions

**James Hodgetts:** conceptualization, data curation, formal analysis, investigation, methodology, project administration, resources, software, visualization, writing – review and editing, writing – original draft. **Germano Gallicchio:** conceptualization, data curation, formal analysis, methodology, resources, software, supervision, visualization, writing – review and editing, writing – original draft. **Shaun Mckiernan:** data curation, methodology, software, writing – review and editing. **Christopher Ring:** conceptualization, methodology, writing – review and editing. **Jennifer Murphy:** conceptualization, writing – review and editing. **Andrew Cooke:** conceptualization, formal analysis, methodology, resources, supervision, visualization, writing – original draft, writing – review and editing.

## Conflicts of Interest

The authors declare no conflicts of interest.

## Supporting information


**Appendix S1:** psyp70415‐sup‐0001‐AppendixS1.zip.

## Data Availability

The data that supports the findings of this study are available in Supporting Information of this article.

## Endnotes

^1^
0° and 360° are equivalent because they denote the same position on the unit circle.

^2^
Full details of the tone and timing parameters are reported in the accompanying software script linked above. Differently from us, the original PAT implementations generated the initial tone at R‐wave + random delay. The demonstration dataset reported below used a 200 ms tone duration, but we have reduced this to 30 ms in the release linked above to enhance temporal precision. All parameters of our implementation are fully customizable.

^3^
Note the vibrations were accompanied by a faint yet audible buzzing sound emitted by the actuator and hence the exteroceptive task can be more precisely considered as a tone to vibration/buzz synchronization exercise. Full details of the tone parameters are reported in the accompanying software script linked above. These parameters are fully customizable.

^4^
Palmer et al. (2025) use the terms phase‐based consistency (for scores mapped to variable heart periods) and delay‐based consistency (for scores mapped to a fixed heart period). Table 1 provides a glossary of our key terms and their equivalents (where appropriate) from previous research.

^5^
The calculation was performed twice. Once using the anticipated period (i.e., the duration between last observed R‐wave prior to the space bar being pressed, and the anticipated next R‐wave calculated based on the mean duration of the three preceding heart periods) and once using the observed period (i.e., the duration between the last observed R‐wave prior to the space bar press and the next observed R‐wave that occurred after the space bar was pressed). The effect of choosing the observed versus the anticipated heart period is explored and discussed in the demonstration section below.

^6^
Although we selected 28‐trials for our protocol, the PAT‐ECG software is programmed to allow up to 45‐trials per recording, thereby providing scope for the experimenter to immediately run additional trials to replace any he deemed invalid from the initial 28‐trial set. The 45‐trial ceiling is configurable and can be expanded if required. In the current dataset, most participants required no replacement trials, and the maximum number of replacement trials added for a single participant was nine.

^7^
We use the terms “interoceptive” and “non‐interoceptive” solely to indicate whether a participant's performance on the PAT‐ECG exceeded (“interoceptive”) or did not exceed (“non‐interoceptive”) the calculated 95th percentile threshold derived from their data. These labels are not intended as diagnostic classifications and should not be interpreted as indicating the overall presence or absence of interoceptive ability. Rather, they reflect performance on a specific cardiac interoceptive accuracy task at a single time point (i.e., during seated rest, in the case of our demonstration dataset).

^8^
It should be noted that in our dataset this individualized approach yielded identical classification outcomes to those generated using a generic threshold, as per Palmer et al. (2025). Accordingly, for the purposes of classifying individuals as interoceptive or non‐interoceptive, the less computationally demanding generic threshold approach appears to be a viable alternative.
